# 1326. Comparison of Positive BioFire® FilmArray® Meningitis Encephalitis (ME) Panels, CSF cultures, CSF Parameters and Inpatient Mortality Among Patients with Bacterial and Fungal Meningitis

**DOI:** 10.1093/ofid/ofad500.1164

**Published:** 2023-11-27

**Authors:** Thein Myint, Marice Ruiz Conejo Castillo, Jaime Soria, Vaneet Arora, Julie Ribes

**Affiliations:** University of Kentucky, Lexington, Kentucky; University of Kentucky, Lexington, Kentucky; University of Kentucky, Lexington, Kentucky; University of Kentucky, Lexington, Kentucky; UK HealthCare, Lexington, Kentucky

## Abstract

**Background:**

The aim of this study was to evaluate the performance of the BioFire® FilmArray® Meningitis Encephalitis panel (MEP) for the detection of bacterial and fungal pathogens compared with cerebrospinal fluid (CSF) cultures and other parameters to determine the predicted value of a positive result (PPV).

**Methods:**

This study is a retrospective chart review of patients with positive MEP from 10/2016–9/2022 at the University of Kentucky. Results of the MEP, culture, cellularity, protein, and glucose were collected using an electronic medical record search. Chart review was performed to collect demographic data, presentation, and test indication if a bacteria or fungus was identified. MEP was considered true positive (TP) if it was confirmed by CSF culture. It was classified a likely TP based on a positive CSF Gram stain, blood culture, other CSF findings consistent with clinical meningitis. It was considered false positive (FP) if the MEP was discordant with CSF and clinical findings. Statistical analysis was performed.

**Results:**

Of the 11,969 panels tested, 132 (1.1%) were positive for bacterial or fungal pathogens. Seventeen positive repeats were excluded. Clinical characteristics, outcomes, and CSF findings for 115 patients with positive MEP for bacterial and fungal organisms are shown in **Table 1**. Results were summarized as TP, likely TP or FP by the pathogen detected (**Table 2**). The majority of results [106(92.2%)] were classified as TP or likely TP whereas 9 (7.8%) were classified as FP. 28/37 (75.7%) of patients with negative CSF cultures received at least one day of antibiotics prior to lumbar puncture. One third of the FP results were *S. agalactiae.* FP were statistically different from those classified as positive for cellularity, glucose and protein **(Table 3).**

**Table 1.**
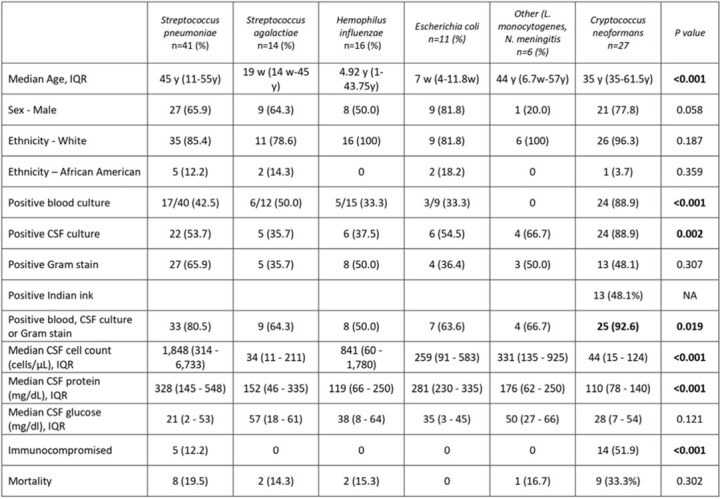
Clinical Characteristics, Outcomes, and CSF Findings of Patients with BioFire® FilmArray® ME panels Positive for Bacterial and Fungal Organisms

**Table 2:**
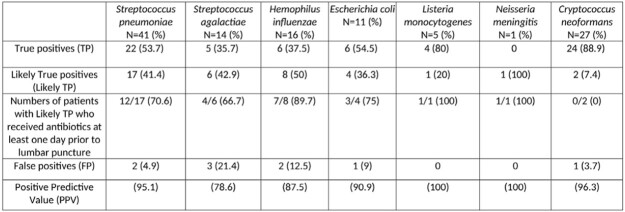
Clinical Correlation of Positive BioFire® FilmArray® Meningitis Encephalitis Panel (MEP) for Bacterial and Fungal Organisms and CSF Parameters

**Conclusion:**

The PPV of MEP ranged from 78.6-100% based on an organism. Three-quarters of negative CSF cultures were likely from a prior antibiotic exposure. FP MEP were most common for *S. agalactiae.* Interestingly, 27.4% (29/106) cases (excluding the FP) would not have had a pathogen definitively identified if MEP was not used. The positive CSF and blood culture rates were higher for cryptococcal meningitis. The median CSF cell count and protein were the highest among patients with *S. pneumoniae* meningitis. Inpatient mortality was uncommon.

**Figure:**
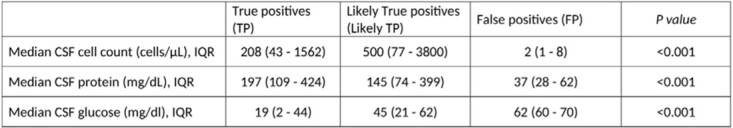

**Table 4.**
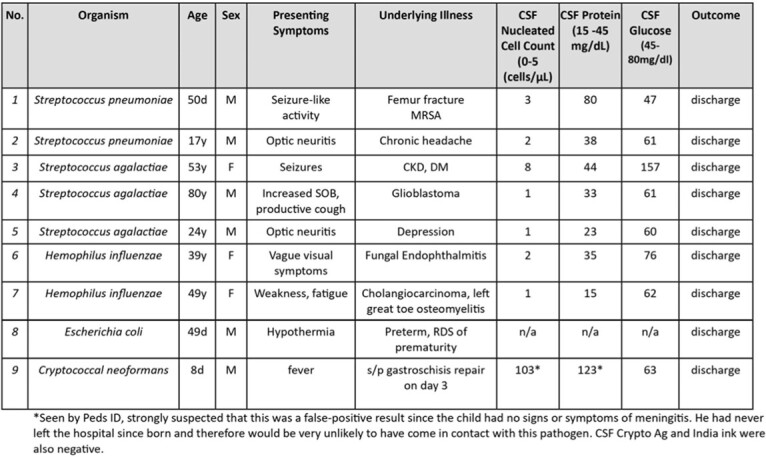
Clinical Characteristics and Outcome of Patients with False Positive Biofire® ME Panel with Negative CSF Gram Stain and Cultures

**Disclosures:**

**All Authors**: No reported disclosures

